# Dataset of parent-child hyperscanning functional near-infrared spectroscopy recordings

**DOI:** 10.1038/s41597-022-01751-2

**Published:** 2022-10-15

**Authors:** Andrea Bizzego, Giulio Gabrieli, Atiqah Azhari, Mengyu Lim, Gianluca Esposito

**Affiliations:** 1 Department of Psychology and Cognitive Science, University of Trento, Rovereto, 38068 Italy; 2 Psychology Program, School of Social Sciences, Nanyang Technological University, Singapore, 639818 Singapore; 3 Neuroscience and Behaviour Laboratory, Italian Institute of Technology, Rome, 00161 Italy; 4 Psychology Programme, School of Humanities and Behavioural Sciences, Singapore University of Social Sciences, Singapore, 599494 Singapore

**Keywords:** Social behaviour, Human behaviour, Social neuroscience

## Abstract

The term “hyperscanning” refers to the simultaneous recording of multiple individuals’ brain activity. As a methodology, hyperscanning allows the investigation of brain-to-brain synchrony. Despite being a promising technique, there is a limited number of publicly available functional Near-infrared Spectroscopy (fNIRS) hyperscanning recordings. In this paper, we report a dataset of fNIRS recordings from the prefrontal cortical (PFC) activity of 33 mother-child dyads and 29 father-child dyads. Data was recorded while the parent-child dyads participated in an experiment with two sessions: a passive video attention task and a free play session. Dyadic metadata, parental psychological traits, behavioural annotations of the play sessions and information about the video stimuli complementing the dataset of fNIRS signals are described. The dataset presented here can be used to design, implement, and test novel fNIRS analysis techniques, new hyperscanning analysis tools, as well as investigate the PFC activity in participants of different ages when they engage in passive viewing tasks and active interactive tasks.

## Abstract (summary)

**Table:** 

Measurement(s)	Blood Oxygen Level-Dependent Response
Technology Type(s)	Functional Near-Infrared Spectroscopy
Factor Type(s)	Parenting Stress • Parental Bonding • Attachment Style
Sample Characteristic - Organism	Homo sapiens
Sample Characteristic - Location	Singapore

## Background & Summary

The term *synchrony* indicates a coordinated interplay of physiological or behavioural signals, and reflects the mutual adjustment of two individuals’ cognitive, emotional, psychophysiological, or behavioral states^1^. Previous works have investigated synchrony between mothers and their children during interpersonal interactions^2–5^. Synchrony in mother-child dyads has been found to contribute to the child’s physiological responses and emotional self-regulation to social stress^6–8^. Analogous to what has been reported for mother-child dyads, brain-to-brain synchrony has been reported for mother-father and father-child dyads^9,10^. Some related studies have since attempted to uncover the nature of synchrony between parent-child dyads in naturalistic tasks. For instance, Liu and colleagues^11^ revealed that synchrony was evident in the middle frontal gyrus and superior frontal gyrus of mother-child dyads when they played a game of Jenga together, indicating their relevance in goal-oriented social decision making and particularly the application of Theory of Mind in the context of cooperative social tasks. Likewise, synchrony has been shown to occur in the dorsolateral PFC^12^, the bilateral PFC and the temporo-parietal areas^4^ of mother-child dyads during cooperative tasks. While the specific cooperative behaviours were not strictly analysed in these cooperative conditions, authors of these studies have proposed that the synchrony observed may be related to oral communication^11^, although Nguyen and colleagues^4^ found an absence of correlations between neural synchrony and ratings of behavioural reciprocity (e.g., turn-taking). Nonetheless, in Reindl and colleagues’ study^12^, it was also shown that synchrony was only observed between parent-child and not stranger-child dyads during the cooperation task, which was theorized to represent an underlying neural mechanism of the emotional connection between the parent and child that is otherwise absent in unrelated dyads. In addition, the PFC is a key brain region that experiences tremendous growth during the preschool age^13,14^, and neuroimaging studies evidence improved functioning of the inferior prefrontal regions^15^ as the child develops. It is interesting to investigate the presence of the parent as a form of social regulation that aids them in emotional regulation following exposure to emotive stimuli, that in turn can be observed as PFC activity^16,17^. Additionally, in neuroimaging studies, the PFC is often the region of interest due to its role in higher-order cognitive processes^18^, such as attentional regulation^19^, emotional perception and modulation^20–22^ and social cognition. As these processes are integral to effective parenting (in terms of attending to and socialising the child to the external environment), the PFC thus represents a key area for investigation in parent-child interactions. Despite the importance of such synchrony, little is known about the synchrony of prefrontal cortical (PFC) activity in mother-child and father-child dyads, and how the magnitude of synchrony is affected by external factors, such as the parent’s level of parenting stress^23^.

In our modern society, there has been a transition from mono-parental parenting, with the mother being the only caregiver, to bi-parental parenting, with spouses actively collaborating with each other^24,25^. This transition has been accompanied by modifications in parents’ brain regions devoted to affect regulation, attention, and cognition. Similarly, in human parents, evidence in support of bio-behavioural synchrony in co-parenting spouses has been reported, such as reciprocal matching of gaze and respiratory patterns^26,27^. Given that synchrony has been found to facilitate both physiological and behavioural correlations between people^28,29^, the possibility that synchrony acts as a central pathway to emotional stability has been theorized. In support of this theory, previous work has revealed that, for instance when partners are “in-sync” with the other’s emotional experience, they are better equipped to support each other^28^.

In addition to their importance in unveiling physiological and neural mechanisms that modulate interpersonal relationships and behaviors, synchrony studies also pose some key questions about the methodological aspects to measure synchrony. In particular, an open issue is the quantification of the extent to which the measured synchrony is due to actual co-presence of the subjects (co-presence-driven synchrony), or to the simultaneous processing of the same stimuli (stimulus-/task-driven synchrony). One of the main strategies to decouple the two components relies on the computation of synchrony between true and surrogate dyads. Surrogate-dyads are created by replacing one of the dyad’s members with another subject randomly selected from the other member’s group^30^. Thus, synchrony measures computed from surrogate-dyads depend only on the stimulus, and can be used as a reference distribution for the amount of stimulus-driven synchrony. Surrogate-dyads’ and true-dyads’ synchrony distributions are then statistically compared to ensure the effect of co-presence on the measured synchrony^31^. However, this strategy is only appropriate for structured, time-controlled experimental designs (for instance, the video sessions of the present dataset). For unstructured experiments (for instance, the play sessions in this dataset), no stimulus-driven synchrony is expected and other methods should be adopted to statistically validate the experimental hypotheses^32^.

To investigate brain-to-brain synchrony, a study employing hyperscanning, the simultaneous recording from multiple individuals, with functional Near-Infrared Spectroscopy signals has been conducted. The PFC brain response signals of mother-child and father-child dyads were recorded while they engaged in two sessions: a) passive video viewing task and b) free play. In addition to fNIRS signals, the dataset also includes dyadic demographic information, the parents’ psychological traits, behavioural annotations for the play session and characteristics of the video stimuli. Given the general dearth of open-source fNIRS signals^33^ and especially of hyperscanning recordings, the dataset can be used to test and develop novel tools for the analysis of fNIRS signals and of brain-to-brain synchrony.

## Methods

### Participants

This collection includes hyperscanning fNIRS recordings from 33 Mother-Child and 29 Father-Child dyads. This study and all its procedures were conducted in accordance with the regulations of the Declaration of Helsinski. The study was approved by the Institutional Review Board of Nanyang Technological University, Singapore (IRB-2018-06-016), and informed consent was obtained from all adult participants prior to the beginning of the experimental session. Children’s assent was requested, while their informed consent was provided by the parents. Parent-child dyads (N = 62; mother-child N = 33, father-child N = 29) were recruited through online forums and social media groups, and voluntarily participated in the study. Inclusion criteria were (1) for the parent, being aged 21 or above with a biological child aged between 34 and 60 months, (2) residing in Singapore, (3) with no known cognitive, visual, or hearing impairments, and (4) dyads must be staying in the same household. A summary of the demographic information on the participants is provided in Table 1.

**Table 1:** Demographic information about study participants.

Type	N	Parent Age (years)			Child Gender		Child Age (months)		
		Average	Min	Max	Male	Female	Average	Min	Max
Father-Child	29	38.1	32	46	18	11	42.2	35	55
Mother-Child	33	34.8	27	46	20	13	42.0	34	55

### Experimental procedure

Each dyad participated in a two-part experimental procedure. In the first part, each dyad was presented with 3 different videos, in random order; in the second part, each dyad engaged in a 10-minute free-play session. This experimental design allowed for the study of different sources and mechanisms of parent-child brain synchrony. During the video viewing portion, subjects were asked to attend to the passive viewing task without interaction. In this context, synchrony was expected to mainly arise from the passive physical presence of each member of the dyad^29,30,34^. During the free play portion, the parent and child were expected to actively interact. In this context, synchrony was expected to be associated with the degree of interaction and engagement^4,35^. For this reason, the free play session was also annotated to quantify the presence and type of different behaviours. Before the experiment, the parent was invited to remotely fill in some questionnaires to quantify their psychosocial personality traits and parenting experiences.

Participants were administered the Parenting Stress Index - Short Form (PSI-SF^36^;), the Attachment Style Questionnaire (ASQ^37^; and the Parental Bonding Instrument (PBI^38^;). The PSI-SF is a self-report questionnaire that measures a parent’s perceived parenting stress level across three subscales, with 12 items in each construct. The Parenting Distress subscale evaluates how confident or restricted parents feel in their parenting role. The Parent-child Dysfunctional Interaction subscale measures the degree to which parents experience satisfaction from their interactions with their child, whereas the Difficult child subscale reflects whether parents believe their child to be difficult or easy to raise. The sum of the three subscales form the total parenting stress index. Another self-assessed questionnaire, the ASQ, evaluates a participant’s adult attachment style with regard to general social relationships. It contains a total of 40 items across five subscales: Confidence, Discomfort with closeness, Relationships as secondary, Need for approval, and Preoccupation with relationships. Following predominant theories of attachment styles which suggest that attachment can be categorised as secure or insecure (avoidant and anxious), the Confident subscale reflects secure attachment, whereas the Discomfort with closeness, Relationships as secondary subscales align with avoidant attachment, and the Need for approval and Preoccupation with relationships subscales parallel anxious attachment. Finally, the PBI is a 25-item measure in which participants retrospectively indicate their perception of their mothers’ and fathers’ parenting behaviours and attitudes. Both maternal and paternal subscales comprise two constructs, Care and Overprotection, where the former indicates the perception of an affectionate and warm parent, whereas the latter captures recollections of a controlling and restrictive parent.

Upon entering the laboratory, a research assistant explained the experimental design to the mother/father, after which the dyad entered the experimental room. The experiment room was made child-friendly to favour the engagement of the children and maximise the compliance to the experimental design. Appropriate sizes of fNIRS caps were selected based on the head circumferences; the optode positions and scalp coupling were adjusted to ensure good signal quality prior to the beginning of data acquisition.

In the first part of the experiment, three videos were shown to the dyad, while the child sat on the parent’s lap. The videos were screened on a laptop that was placed at the centre of the table at a distance of 40 cm from the dyad. Once the video and fNIRS data-recording started, the experimenters exited the experiment room. Each video lasted 1 minute with an inter-stimulus interval of 10 seconds. During this interval, a fixation point was shown on a black screen. After the end of the last video, the experimenters entered the room and stopped the fNIRS recording.

In the second part of the experiment, a table with preschool-aged toys^39,40^ was placed in front of the dyad. In this set-up, the parent and the child sat next to each other. The positions of the table, as well as of the toys, were standardised across participants. Parents were then instructed to play with their child for a total of 10 minutes. Before the start of the play session, fNIRS acquisition was restarted after checking again the quality of the signals. Upon restarting the fNIRS signal recording, the experimenters exited the experimental room. The play session was recorded in .MOV format using a Sony Handycam camcorder mounted on a tripod about 2 meters away from the dyads. Video recordings were used for an offline annotation of different types of target behaviours. At the end of the play session, participants were debriefed, thanked and remunerated.

### fNIRS data acquisition

**Fig. 1:**
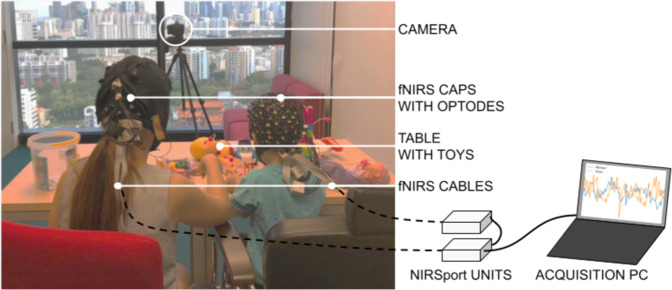
Schematic representation of experimental setup during the play session.

PFC activity was recorded via hyperscanning by means of two NIRSport devices (NIRx Medical Technologies LLC, sampling rate: 7.81 Hz, wavelengths: 760 nm and 850 nm; see Fig. 1).

**Fig. 2:**
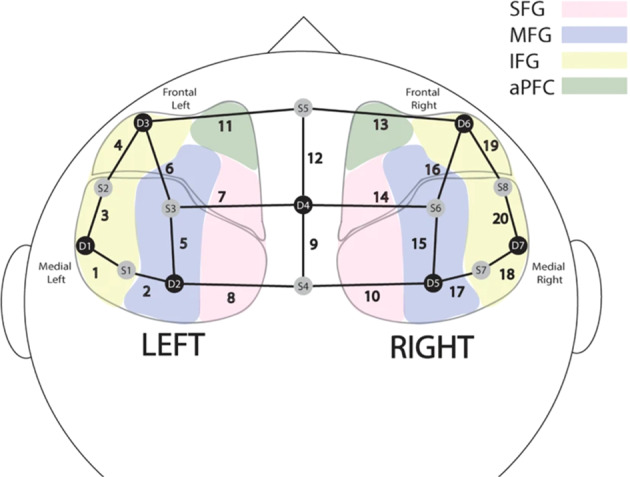
Visual representation of the position of sources and detectors and of generated channels, with respect to the superior frontal gyrus (SFG), middle frontal gyrus (MFG), inferior frontal gyrus (IFG), and anterior prefrontal cortex (aPFC). Figure from^34^.

The imaging montage is made up of 8 sources and 7 detectors, with a maximum of 3 cm inter-optode distance, resulting in 20 different channels. Montage setup and signals were recorded using NIRStar (v15.2, Windows 64 bit), a MATLAB based software provided by NIRx Medical Technologies LLC to acquire and record signals from NIRX devices. A visual representation of the montage is reported in Fig. 2. The PFC region was selected in this study based on previous evidence in the literature that suggest that biobehavioural synchrony emerges in the PFC of parent-child during coordinated interactions.

### Video stimuli

Three 1-min long video stimuli conveying different emotional valence, drawn from three different animated movies and cartoons (“Brave”, “Peppa Pig”, and “The Incredibles”) were selected for presentation. 1-minute blocks were used in this experiment to ensure that child participants were given sufficient time to process emotional information in the movie clips being screened to them. A 10 s inter-stimulus interval was a compromise which allowed hemodynamic responses to be re-stabilised while ensuring that child participants did not grow restless from viewing the fixation cross, thus remaining compliant in the study.

Stimulus visual complexity was measured using the python package pyaesthetics^41^ on still images extracted from the selected videos at 12 frames per second (FPS) using FFmpeg (v. 3.4.4, Linux 64 bit). The fundamental frequency and audio intensity of the audio tracks was estimated using Praat (v. 6.0.46, Linux 64 bit). The emotional valence of the three videos was evaluated microanalytically by means of 60 ratings sampled at a frequency of 1 per second.

Behavioural measures were not coded for during movie-viewing as the primary aim of this phase was to collect fNIRS signals while dyads engaged in a passive joint activity together, which did not require active social interaction. However, this might pose as a limitation as even subtle changes in behaviours, such as parental attempts to decrease or increase proximal distance to the child, could greatly assist in making meaningful interpretations of the synchrony data.

### Behavioural coding

Video recordings from the free play sessions were used to annotate target behaviours which focused on presence of interaction in terms of motor activities and emotion communication. Due to the sensitive nature of the data, the recordings cannot be publicly shared and are therefore not included in the dataset, but they might be privately accessed with prior specific research agreements. The interested reader should contact the corresponding author. To annotate the behaviours, two research assistants proceeded with microanalytic coding of the videos using the Solomon Coder software (Version: 22 March 2017) at a rate of 7.81 Hz. Target behaviours included actions or interactions with the toys (e.g. passing, picking up or holding a toy, squeezing the ball) and emotional behaviours and interactions between the two members of the dyad (e.g. engaging in play with the same object, laughing or smiling, mutual gaze). In addition, the Emotional Availability Scales (EAS)^42^ were used to assess dyadic interactions between the parent and the child. The EAS consists of six subscales: Adult Sensitivity, Adult Structuring, Adult Non-intrusiveness, Adult Non-hostility, Child Responsiveness to the adult, and Child Involvement of the adult. The 10-min free play session was divided into 10 non-overlapping portions of 1-min and each subscale was rated for each portion.

### Parenting measures

Before the experiment, parents were asked to complete several questionnaires to assess parenting-related psychosocial traits.

Parenting stress was assessed using the Short Form (PSI-SF) of the Parenting Stress Index (Fourth Edition), a diagnostic tool designed to measure parents’ perceived stress^36,43,44^. In addition to the Parenting Stress Index (PSI) the following subscales were reported: Parenting Distress, Parent-Child Dysfunctional Interaction, and Difficult Child.

Parenting behaviors and attitudes were assessed using the Parental Bonding Instrument (PBI)^38^. The PBI aims to retrospectively measure parental styles as perceived by the child, based on how adults remember their parents during their first 16 years. The PBI is composed of two scales: Overprotection and Care, which are assessed for both parents (mother and father).

Finally, parent’s attachment style in regard to general relationships was measured using the Attachment Style Questionnaire (ASQ)^45^. The ASQ is composed of five scales: Confidence, Relationships as Secondary, Need for Approval, Discomfort with Closeness, and Preoccupation with Relationships^46^.

For more detailed information regarding the questionnaires, please refer to the above Experimental procedure subsection.

## Data Records

The dataset is available online on the Data Repository of the Nanyang Technological University (DR-NTU Data)^47^. The dataset is first organized by type of session (video and play) then by type of dyad (mother-child and father-child). FNIRS data of each dyad are organized into folders named using the dyad identifier, and then by subject (parent and child). Finally, each subject folder includes the files with raw data and acquisition metadata, created by the NIRStar software.

In particular, the raw fNIRS signals are stored into two files (extensions:.wl1 and .wl2) that record the reads of the photo-diodes at the two wavelengths used in fNIRS. Information about optode positioning and montage, and acquisition settings is stored into three other files (probeInfo.mat, config.txt, and.hdr). For the video session, the information about the onset timestamps is stored in the Conditions subfolder; additionally, information about the video stimuli (visual complexity at a rate of 12 frames per second in _VC.csv files, as well as average video complexity, main frequency and emotional valence in the metadata_video.csv file) is provided.

For the play session, the dataset includes the annotations of the target behaviours (behavioural_mothers.csv and behavioural_fathers.csv), the EAS ratings EAS.csv and two documents with the details about the annotated behaviours (MotorParameters.docx and EmotionParameters.docx).

The dataset also includes demographic information about the dyads (demographics.csv) and the results of the questionnaires on parenting (parenting.csv).

## Technical Validation

The montage used for fNIRS data acquisition relies on the international 10–20 system employed in EEG recordings, as previously done in other studies^48^. More specifically, a 20-channel montage that covers the superior frontal gyrus (SFG), the middle frontal gyrus (MFG), the inferior frontal gyrus (IFG) and the anterior prefrontal cortex (aPFC) was used. A visual representation of the montage is reported in Fig. 2. The same montage was employed for both adult and children participants. Cap sizes were selected according to the head circumference of the participants, and positioned according to reference anatomical loci. The procedure allowed for providing an optimal contact between the surface of the probes and the scalp, and for minimising the likelihood of slips. Sources (N = 8) and detectors (N = 7) were manually placed after the cap had been worn. Although slight variations in the source-detector separation distances across child and adult caps was present, these never exceeded the maximum optimal distance of 3 cm. The validity of collected fNIRS data was ensured by trained experimenters before starting the acquisition. In particular, the quality of the fNIRS signals was checked using the visual inspection tool provided by the NIRStar software. The tool showed which fNIRS channels had poor data quality so the experimenters could adjust the coupling between the optodes and the scalp before starting the acquisition.

During the signal quality checking procedure, an automatic calibration of the internal signal acquisition parameters (e.g. amplification gain) is performed by the instrument, for each subject and channel independently. This step allows for the partial addressing of physiological differences between subjects and, more importantly, between adults and children. The validity of the collected signals was assessed before each experimental session.

This procedure, however, cannot prevent the potential loss of signal quality during the experiment due to movements of the subjects^49^. For this reason, it is recommended that any fNIRS signal processing pipeline includes a quality check step, to reject the channels that show poor data quality or presence of artifacts due to movements^50,51^.

We report the overall signal quality of the dataset in Table 2. Specifically, we applied a recently automated approach based on Deep Learning^52^ to obtain the portion of signal with good quality for each channel and subject, in percentage. Then we averaged the measure across the dyads, by type of dyad, session and member group. Overall, on average, more than 75% of the signal lengths had good quality. However, Channel 5 particularly had lower quality signals. We note, however, that this is the baseline quality of raw data; signal processing and motion artefact removal procedures are expected to improve the quality.

**Table 2:** Portion of signal with good quality in percentage.

Parent	Session	Member	Mean	SD	Min [channel]	Max [channel]
Father	Play	Child	89.1	6.5	71.4^5^	98.3^17^
		Parent	88.1	11.3	63.4^5^	99.9^4^
	Video	Child	82.2	13.7	41.3^5^	99.7^17^
		Parent	79.6	15.9	42.1^5^	97.8^4^
Mother	Play	Child	86.4	13.6	60.2^15^	100.0^19^
		Parent	88.3	10.5	64.0^5^	99.5^6^
	Video	Child	75.3	20.3	36.5^15^	97.9^4^
		Parent	81.9	13.6	51.6^5^	98.3^13^

Values are aggregated across all dyads, by type of dyad, session and member group. Mean and SD computed across the 20 channels are reported, with minimum and maximum value and respective channels.

Regarding the validity of the behaviour annotations, inter-rater agreement between the two coders was calculated using the irr package. An inter-rater agreement of at least 80% was achieved across the coders.

This dataset has been used in several studies that investigated synchrony and brain response in parent-child dyads. Mothers’ parenting stress^23^ has been found to be associated with reduced brain-to-brain synchrony; and an exploratory study suggests that mothers’ attachment anxiety might also influence brain coupling^53^. Synchrony in father-child dyads was also found during the video stimulation session^54^, possibly associated with father age and parenting experience. When analysing group-level neural activation between mothers and children, asymmetric brain response was found in mothers compared to children when mutual gaze occurred during the play session^55^, where mothers demonstrated PFC deactivation while children showed an activation, implying divergent PFC mechanisms even though dyads are engaged in symmetrical behaviours such as mutual gaze. Additionally, the presence of parent and emotional valence of the video stimuli have been found associated with the brain response of children^56^. Finally, the dataset has also been used to develop signal processing algorithms and quantitative methods to compute brain coupling^32^.

## Usage Notes

The dataset is released under the Creative Common license (CC-BY-NC) for non-commercial use only. To read the fNIRS data, specific software can be used, for instance homer2^57^, mne^58^ or custom scripts able to read MATLAB datafiles and text files. Raw fNIRS signals are are provided to allow for the highest flexibility of developing and testing signal processing algorithms. Raw signals need to be processed to remove signal components and noise not associated with the functional activity of the brain. A typical signal processing pipeline should include the following steps: signal quality check, removal of motion artifact and physiological components, filtering, and conversion to Oxy and DeOxy signals. While the definition of an optimal standardized procedure is still an open research question, some works collect the main approaches and algorithms^59–61^ and can be used as a reference to design the signal processing pipeline. Many software and toolboxes are available to implement the signal processing pipelines (see Almajidy and colleagues’ review^62^, including, but not limited to, homer^57^, NirsToolbox^63^, nirs-spm^64^ and mne-fnirs^60^.

The dataset is a valuable resource that can be exploited for many different purposes. First, given the lack of freely available hyperscanning recordings, the data can be employed to develop and test appropriate methodological approaches to study inter-personal brain coupling in this specific context. Synchrony measures can be applied, based on several signal similarity measures and using different statistical approaches^31,32,65^.

Second, the dataset offers a high number of individual fNIRS recordings, and featured two important characteristics that make it a precious independent dataset to evaluate and calibrate new signal processing algorithms. First, compared to typical event-based or block-based experimental designs used in fNIRS studies, data from the play session are collected in an unstructured experimental scenario, where the subjects were free to move and interact. They are therefore relevant to evaluate algorithms for the detection and correction of movement artifacts. Second, this dataset includes signals collected from children, that represent a special category of subjects^66^.

Finally, the data allows for further analysis of different demographics and psychological traits, brain activity and parent-child synchrony and their relations with parental variables such as parenting stress.

## Data Availability

The dataset presented in this manuscript includes raw data that have not been processed by any script or software-based procedure.
